# Assessing the impact of fasting condition and storage time on plasma Amyloid‐β 42 and 40, pTau181, Nf‐L and GFAP measurements

**DOI:** 10.1002/alz.091245

**Published:** 2025-01-09

**Authors:** Helena Blasco‐Forniés, Javier Torres‐Torronteras, Paula Ortiz‐Romero, Armand González Escalante, Carolina Minguillón, Federica Anastasi, Esther Jiménez‐Moyano, Marc Suárez‐Calvet, Marta del Campo

**Affiliations:** ^1^ Barcelonaβeta Brain Research Center (BBRC), Pasqual Maragall Foundation, Barcelona Spain; ^2^ Hospital del Mar Medical Research Institute (IMIM), Barcelona Spain; ^3^ Hospital del Mar Research Institute (IMIM), Barcelona Spain; ^4^ Universitat Pompeu Fabra, Barcelona Spain; ^5^ Centro de Investigación Biomédica en Red de Fragilidad y Envejecimiento Saludable (CIBERFES), Madrid Spain; ^6^ Barcelonaβeta Brain Research Center (BBRC), Barcelona Spain; ^7^ IMIM (Hospital del Mar Medical Research Institute), Barcelona Spain; ^8^ Centre for Genomic Regulation (CRG), Barcelona Institute of Science and Technology (BIST), Barcelona Spain; ^9^ Servei de Neurologia, Hospital del Mar, Barcelona Spain; ^10^ Departamento de Ciencias Farmacéuticas y de la Salud, Facultad de Farmacia, Universidad San Pablo‐CEU, CEU Universities, Madrid Spain

## Abstract

**Background:**

Blood biomarkers are essential in identifying Alzheimer's disease (AD) pathology. To ensure their clinical use, it is crucial to understand pre‐analytical factors such as fasting conditions and long‐term storage at ‐80°C. This study evaluates the effect of these factors on plasma biomarker concentrations for detecting AD pathology and neurodegeneration.

**Methods:**

Plasma biomarkers were measured in participants from the ALFA cohort using N4PE (Amyloid‐β [Aβ]40, Aβ42, Nf‐L, GFAP) and the pTau181 v2.1 advantage kits on Simoa HD‐X analyzer (Quanterix). To study the impact of fasting conditions, plasma samples from the same 16 participants in fasting and non‐fasting conditions were analysed. Aβ positivity in these participants was determined using CSF Aβ42/40 ratio. To assess long‐term storage at ‐80°C, we measured plasma biomarkers in samples stored at ‐80°C for 10 years (n=2339) and one year (n=381) from different participants. We compared differences on biomarker levels across groups, adjusting for age, sex and APOE‐ε4 genotype.

**Result:**

Plasma concentrations of Ab40 and Aβ42 were lower in samples obtained under fasting conditions than non‐fasting ones independently of their amyloid status. However, this effect was not observed in the plasma Aß42/40 ratio. No differences in the plasma concentration of GFAP, Nf‐L or pTau181 were detected between fasting and non‐fasting conditions. The performance of the different markers to discriminate amyloid positivity was similar across fasting conditions. We observed no differences on pTau181 levels between long‐ and short‐term storage at ‐80°C. However, we observed higher levels of Ab40, Ab42 and Ab42/40 (mean difference: 9.7%, 16.8% and 7.0%, respectively) and lower levels of GFAP (32.1%) and Nf‐L (13.9%) in samples stored for 10 years.

**Conclusion:**

Plasma pTau181, a key predictor of amyloid pathology, remains unaffected by fasting and long‐term storage. However, fasting conditions influenced the measurements of Aβs, which was mitigated by considering the Aβ42/40 ratio. Notably, long‐term storage at ‐80°C may influence the concentration of Abs, GFAP, and Nf‐L, underpinning the need to consider storage time on plasma biomarker analyses. Future studies should include larger data sets for fasting conditions and evaluate additional factors that may influence the effect of storage time on biomarker levels.

